# *De novo* transcriptome and expression profile analyses of the Asian corn borer (*Ostrinia furnacalis*) reveals relevant flubendiamide response genes

**DOI:** 10.1186/s12864-016-3431-6

**Published:** 2017-01-05

**Authors:** Li Cui, Changhui Rui, Daibin Yang, Zhenying Wang, Huizhu Yuan

**Affiliations:** 1 Key Laboratory of Integrated Pest Management in Crops, Ministry of Agriculture, Institute of Plant Protection, Chinese Academy of Agricultural Sciences, Beijing, 100193 People’s Republic of China; 2 State Key Laboratory for Biology of Plant Diseases and Insect Pests, Institute of Plant Protection, Chinese Academy of Agricultural Sciences, Beijing, 100193 People’s Republic of China

## Abstract

**Background:**

The Asian corn borer (ACB), *Ostrinia furnacalis* (Guenée), has become the most damaging insect pest of corn in Asia. However, the lack of genome or transcriptome information heavily hinders our further understanding of ACB in every aspect at a molecular level and on a genome-wide scale. Here, we used the Ion Torrent Personal Genome Machine (PGM) Sequencer to explore the ACB transcriptome and to identify relevant genes in response to flubendiamide, showing high selective activity against ACB.

**Results:**

We obtained 35,430 unigenes, with an average length of 716 bp, representing a dramatic expansion of existing cDNA sequences available for ACB. These sequences were annotated with Non-redundant Protein (Nr), Gene Ontology (GO), Clusters of Orthologous Groups (COG) and Kyoto Encyclopedia of Genes and Genomes (KEGG) to better understand their functions. A total of 31 cytochrome P450 monooxygenases (P450s), 27 carboxyl/cholinesterases (CCEs) and 19 glutathione S-transferases (GSTs) were manually curated to construct phylogenetic trees, and 25 unigenes encoding target proteins (acetylcholinesterase, nicotinic acetylcholine receptor, gamma-aminobutyric acid receptor, glutamate-gated chloride channel, voltage-gated sodium channel and ryanodine receptor) were identified. In addition, we compared and validated the differentially expressed unigenes upon flubendiamide treatment, revealing that the genes for detoxification enzymes (P450s and esterase), calcium signaling pathways and muscle control pathways (twitchin and tropomyosin), immunoglobulin (hemolin), chemosensory protein and heat shock protein 70 were significantly overexpressed in response to flubendiamide, while the genes for cuticular protein, protease and oxidoreductase showed much lower expression levels.

**Conclusion:**

The obtained transcriptome information provides large genomic resources available for further studies of ACB. The differentially expressed gene data will elucidate the molecular mechanisms of ACB in response to the novel diamide insecticide, flubendiamide. In particular, these findings will facilitate the identification of the genes involved in insecticide resistance and the development of new compounds to control the ACB.

**Electronic supplementary material:**

The online version of this article (doi:10.1186/s12864-016-3431-6) contains supplementary material, which is available to authorized users.

## Background

The Asian corn borer (ACB), *Ostrinia furnacalis* (Guenée) (Lepidoptera: Crambidae) is the most destructive lepidopteran pest of corn in Asia, particularly in China and the Philippines. ACB larvae damage corn by attacking the new leaves, ears and cobs; these pests also bore into the stalks and cobs of corn [1]. The damage manifested by ACB infestation constitutes a major constraint on agriculture in corn-producing countries worldwide. Yield losses caused by the ACB are estimated at 10 to 20% in an ordinary year but may be more than 30% or may even result in no harvest in an outbreak year [2]. Moreover, the corn borer is considered an aggravating factor for the epidemiology of *Fusarium* ear rot in maize, and insect damage to ears can increase the fumonisin contamination of kernels [3, 4]. Novel insecticides with high selective activity are preferred to control this insect pest. Recently, two classes of diamide chemicals have emerged as novel insecticides targeting insect RyRs: the phthalic diamides, including flubendiamide [5, 6], and the anthranilic diamides, including chlorantraniliprole and cyantraniliprole [7]. These diamides have potent species-specific insecticidal activity against a range of lepidopteran pest species and can elicit intracellular Ca^2+^ release from the sarcoplasmic reticulum or endoplasmic reticulum [8]. Yang et al. reported that chlorantraniliprole provided excellent control efficacy against second-generation ACB and was relatively safe to the spider *Xysticus ephippiatus*. In addition, we previously demonstrated that flubendiamide also showed high efficacy against the ACB. Consequently, these diamide insecticides are suitable for integrated pest management programs for the regulation of the ACB [3]. However, the molecular mechanisms of the ACB in response to diamide insecticides are not clearly understood.

Despite the insect’s importance in agricultural production, the sequence information available for the ACB lies in contrast to the economic importance of this pest. The current understanding of this pest has largely been hindered by the lack of thorough genetic information. Until recently, no complete picture has been achieved, even of specific gene families. For example, cytochrome P450s (CYPs) have been highly recognized as a supergene family, with 36 to 180 genes in insect genomes [9, 10], while only one CYP gene with a full-length coding region was accessed for the ACB in GenBank. This scarcity of genetic information for the ACB has also resulted in a paucity of genetic studies and integrated theories for understanding the basic biology of this pest.

The aim of the present study was to use PGM sequencing to produce a *de novo* transcriptome of the ACB as a resource for current and future studies of this pest species. This resource was subsequently used as a reference to provide insights into insecticide-related changes in gene expression in flubendiamide-treated ACB and to identify a broad range of genes encoding the target proteins and detoxification enzymes of insecticides currently in use for ACB control.

## Results

### RNA-Seq and sequence assembly

**Fig. 1:**
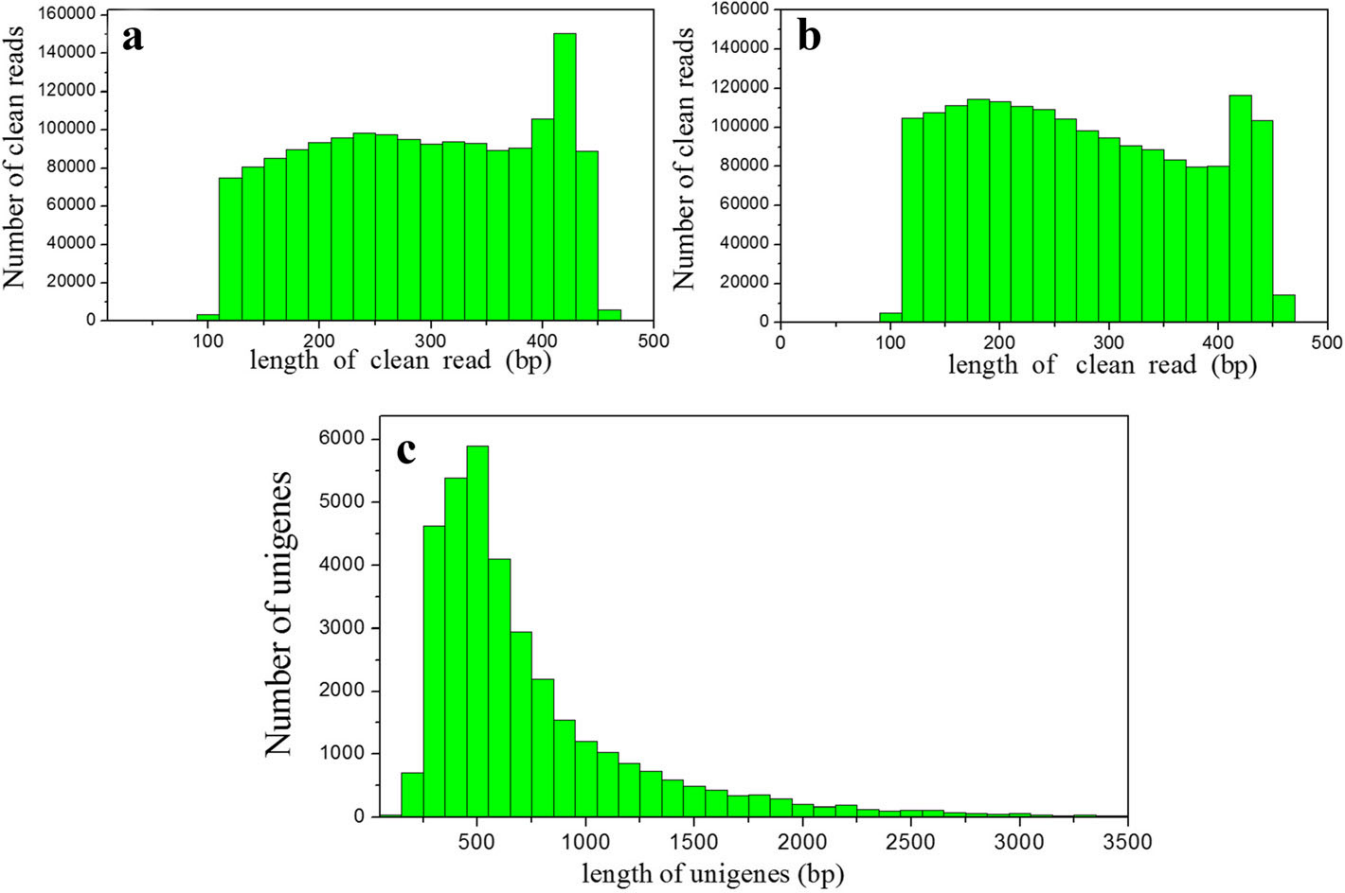
Frequency distribution of PGM sequencing read length for the control library (**a**), the flubendiamide-treated library (**b**) and the assembled unigenes (**c**)

When two ACB libraries (Control strain feed on a semi-artificial diet without insecticide treatment and flubendiamide-treated strain) were sequenced using the PGM platform, the output was 549.4 and 472.5 Mb. After trimming the adaptor sequences and removing the low-quality reads, 1,625,223 and 1,732,688 clean reads were generated from the control and flubendiamide-treated libraries, with average sequence lengths of 279 and 265 bp, respectively. The clean reads of the two libraries (control and flubendiamide-treated) were assembled into 31,059 and 31,478 unigene sequences, with average unigene lengths of 759 and 756 bp, respectively. A summary of the PGM sequencing and assembly is presented in Table 1, and the length distributions for the clean reads and unigenes are presented in Fig. 1.

**Table 1:** An overview of the sequencing and assembly process of the ACB

	Control library	Flubendiamide-treated library
Clean reads	1625223	1732688
Clean read mean length	279 bp	265 bp
Unigenes	31059	31478
Unigene mean length	759 bp	756 bp

### Analysis of the transcripts from the Asian corn borer

**Fig. 2:**
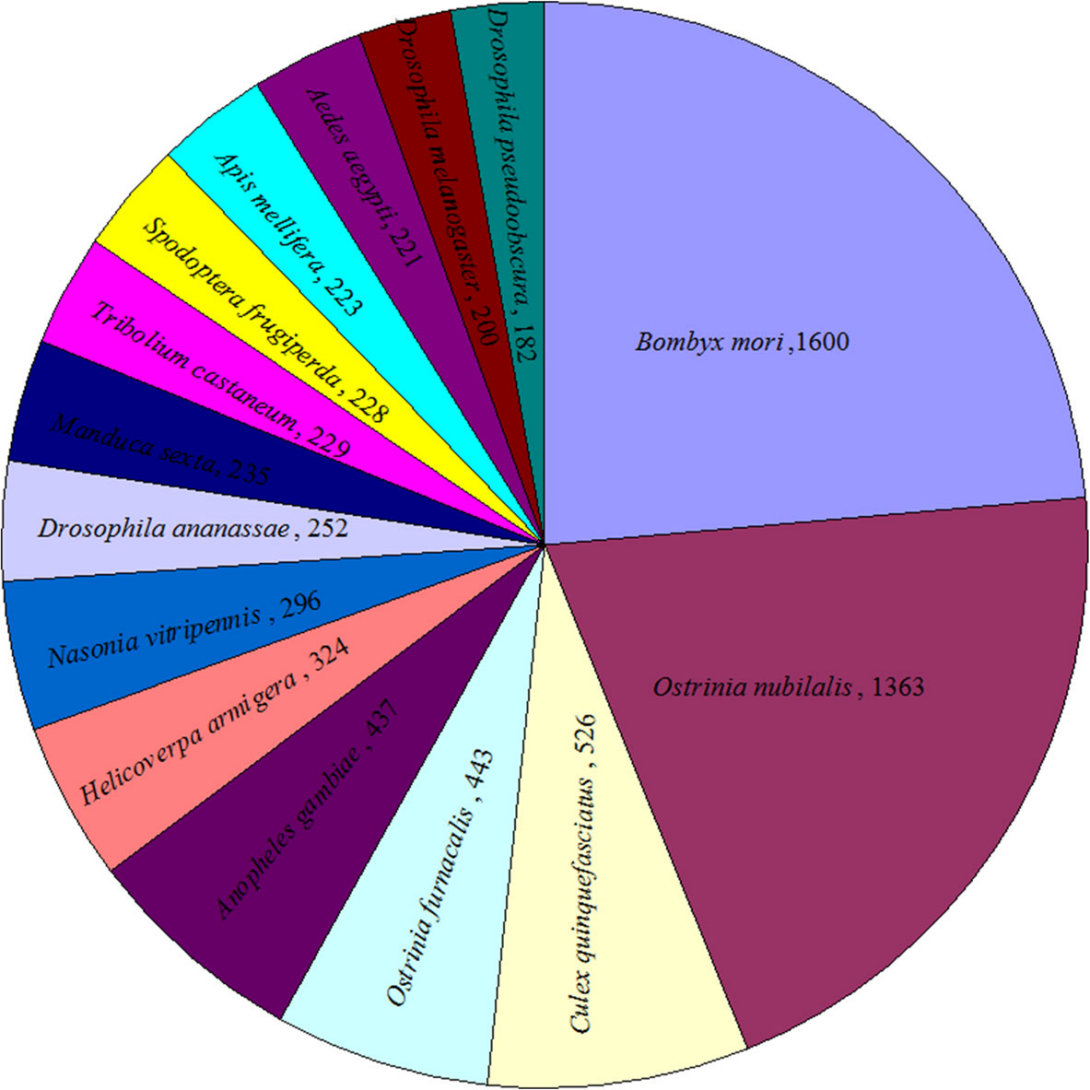
Top 15 insect species with significant BLASTn hits. All ACB unigenes were used in BLASTn searches against the GenBank entries. The significant hits with *E*-values ≤ 1.0E^−5^ for each query were grouped according to species, and the numbers of unigenes with significant homology are indicated after the species name

BLASTx and BLASTn were used to compare each ACB transcript with a cut-off *E*-value of 1.0E^−5^ against GenBank entries. A total of 19,422 transcripts (55%) had BLASTx hits in Non-redundant Protein (Nr) databases, and 11,004 transcripts (31%) had BLASTn hits in Non-redundant Nucleotide Sequence (Nt) databases. Some of the ACB transcripts were homologous to those from more than one species, but in general, most ACB transcripts were homologous to Lepidoptera species, accounting for 4392 hits among the 11,004 BLASTn hits, including 1600 hits (14.5%) to *Bombyx mori* entries, 1363 hits (12.4%) to *Ostrinia nubilalis*, and 443 hits (4.0%) to *Ostrinia nubilalis*. The second highest hits were to Dipteran species, with 526 hits to *Culex quinquefasciatus* and 437 and 252 hits to the *Anopheles gambiae* and *Drosophila ananassae*, respectively. The top 15 insect species with significant BLASTn hits are shown in Fig. 2.

### Unigene function annotation

**Fig. 3:**
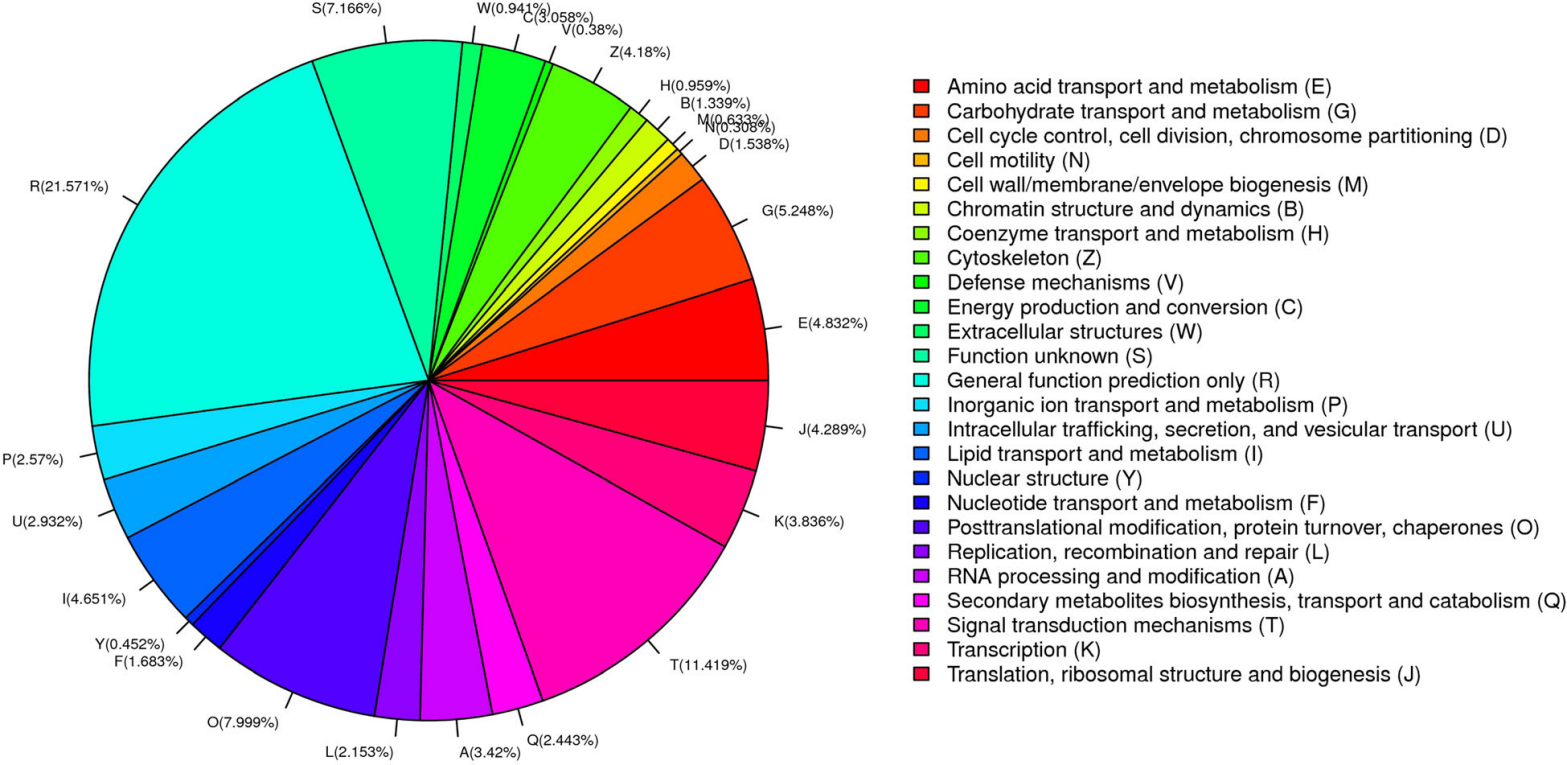
Clusters of Orthologous Groups (COG) classification

To analyze the putative protein functions, the Clusters of Orthologous Groups (COG) database was used to predict and classify the potential functions of the identified unigenes. Using sequence homology, 15,188 unigenes (43%) were annotated and divided into 25 specific categories (Fig. 3). Among the COG classifications, the cluster of general function (21.57%) was the largest, followed by signal transduction mechanisms (11.42%) and posttranslational modification, protein turnover, and chaperones (8.00%). The categories nuclear structure (0.45%), defense mechanisms (0.38%) and cell motility (0.31%) represented the smallest groups.

**Fig. 4:**
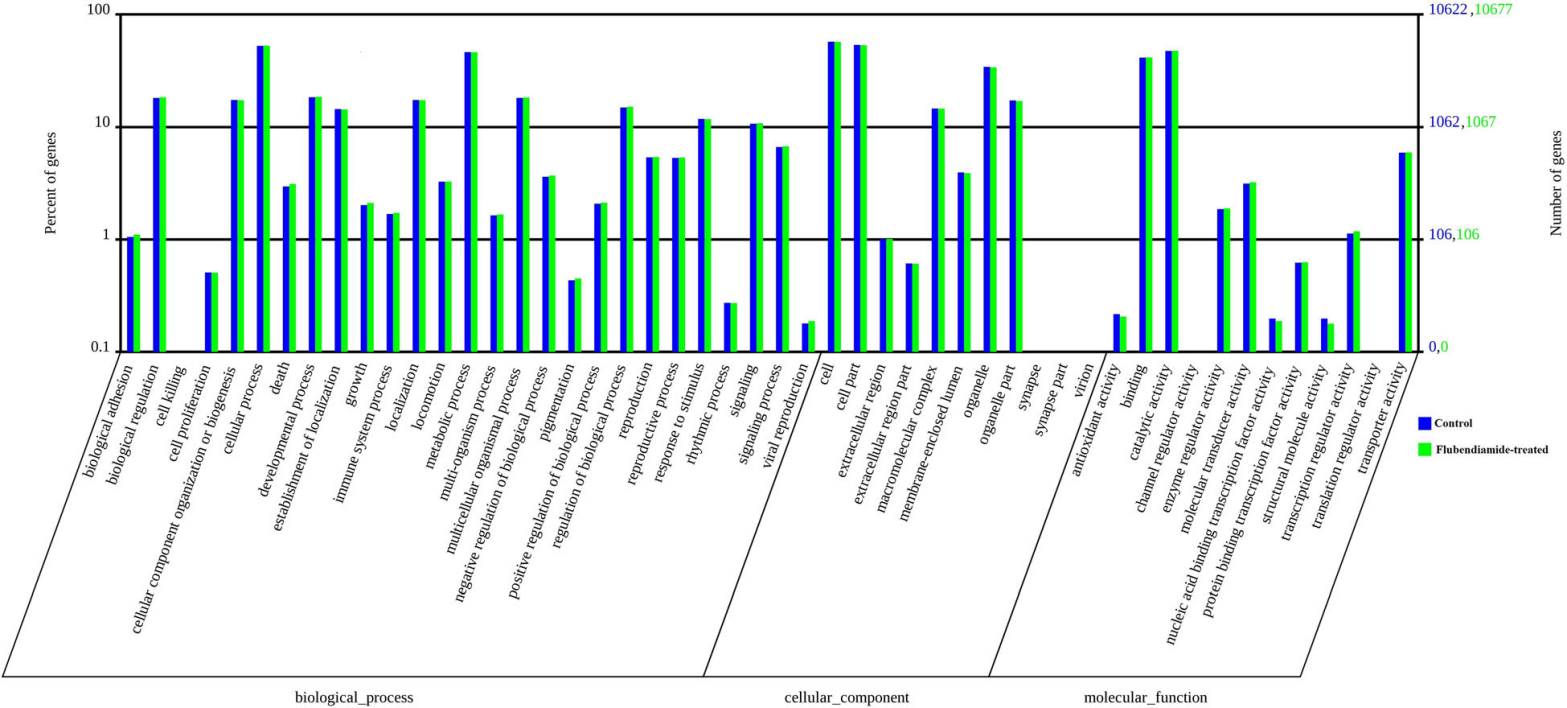
Gene Ontology classification of assembled unigenes. The right y-axis indicates the number of genes in a category, whereas the left y-axis indicates the percentage of a specific category of genes in that main category

We used the Gene Ontology (GO) database to obtain functional annotations of the ACB unigenes [11]. A total of 11,349 transcripts (32%) were assigned to at least one GO term in three categories: biological processes, cellular component and molecular function (Fig. 4). The three categories were further classified into 50 functional subcategories (Additional file 1), among which cell and cell part in the cellular component, catalytic activity and binding in the molecular function, and cellular processes and metabolic processes in the biological processes represented the major subcategories, while fewer than 10 unigenes were observed in translation regulator activity (8), synapse (7), synapse part (3), virion (3), cell killing (2), and channel regulator activity (1). Some unigenes were assigned to multiple GO term categories, while others could not be assigned to a given GO term. The biological process terms were primarily associated with cellular processes, such as proteolysis, carbohydrate metabolic processes and oxidation-reduction utilization. Similar compositions and distributions of unigenes assigned to GO terms have been reported in transcriptomics descriptions from other insects [9, 12].

### Unigene metabolic pathway analysis

The unigene metabolic pathway analysis was conducted using the Kyoto Encyclopedia of Genes and Genomes (KEGG) annotation system. A total of 8512 unigenes (24%) were mapped to 186 KEGG pathways (Additional file 2). The most enriched pathways included metabolic pathways, biosynthesis of secondary metabolites, purine metabolism, focal adhesion, phagosome, spliceosome, and protein processing in the endoplasmic reticulum and the ribosome. These annotations provide a valuable resource for the investigation of specific processes, functions and pathways in the ACB.

### Transcripts encoding detoxification enzymes and insecticide targets

The known mechanisms underlying insecticide resistance in the ACB include decreased penetration, increased detoxification and target insensitivity. To screen genes that may evolve insecticide resistance, we mined the current transcriptomics data to identify unigenes encoding insecticide targets or detoxification enzymes. A number of sequences homologous to detoxification enzymes (P450s, CCEs and GSTs) and insecticide targets were identified.

**Fig. 5:**
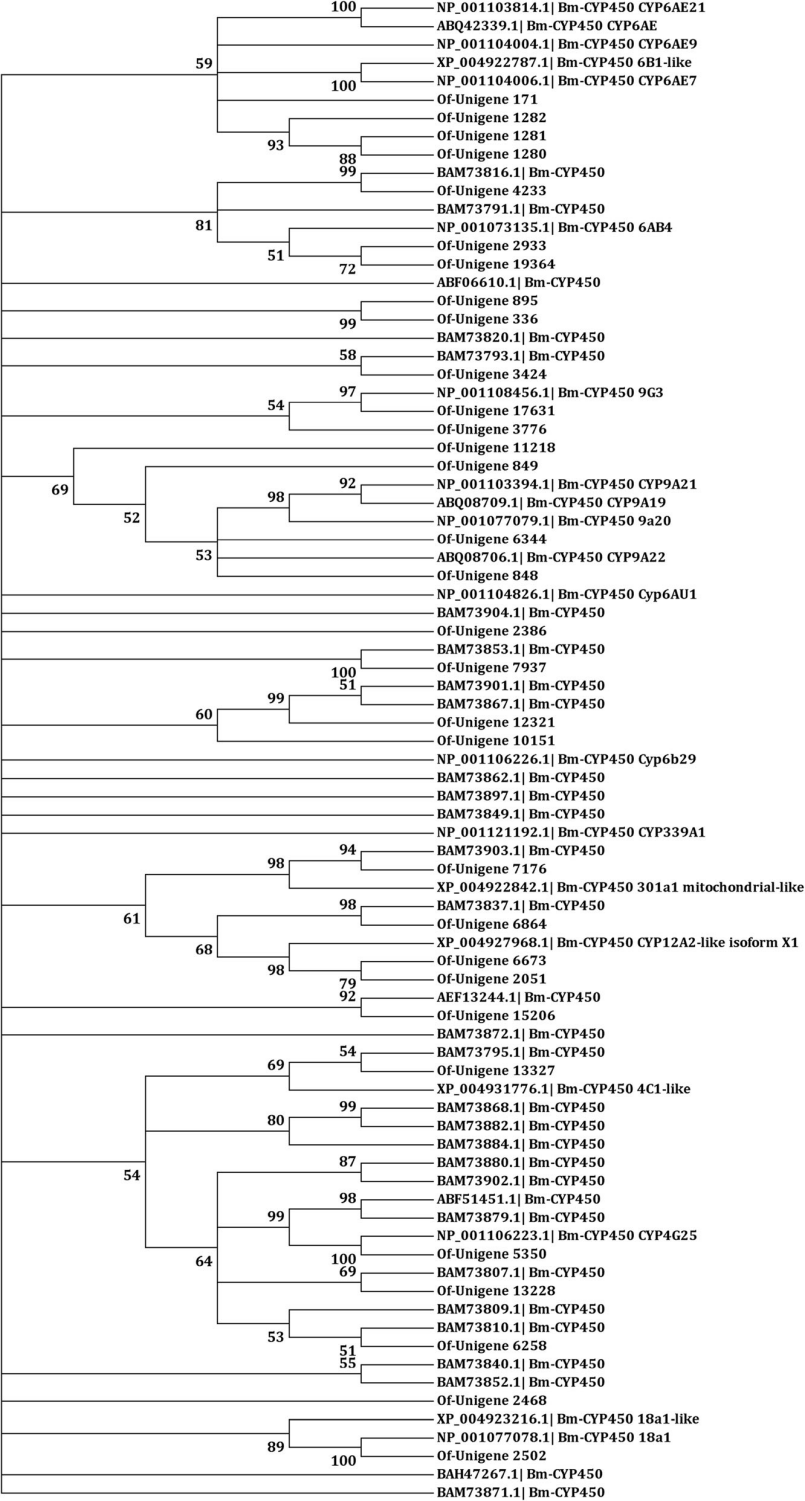
Neighbor-joining phylogenetic analysis of the cytochrome P450s from *Ostrinia furnacalis* (Of) and *Bombyx mori* (Bm). The tree was constructed from the multiple alignments using MEGA5.0 software and generated with 1000 bootstrap trials using the neighbor-joining method. The numbers indicate the bootstrap confidence values obtained for each node after 1000 repetitions. Positions containing alignment gaps and missing data were eliminated with pairwise deletion

A total of 174 P450-related unigenes were identified from the Nr annotation of the ACB transcriptome. After manually removing allelic variants of the same P450 gene and unigenes with short open reading frames (ORFs) <600 bp, the remaining 31 P450 unigene sequences (Additional file 3) were used to construct a phylogenetic tree. Based on the closest BLAST hits in the NCBI Nr database and the phylogenetic analysis with P450 genes from *B. mori*, the P450s were assigned to one of four CYP clans: CYP2, CYP3, CYP4 and the mitochondrial clan. CYP3 ranked as the largest clan, consisting of 11 members belonging to the CYP6 family and seven genes belonging to the CYP9 family. The CYP4 clan included five P450s from the CYP4 family, and the remaining P450 genes belonged to the mitochondrial (6) and CYP2 (1) clans, which might be involved in the ecdysteroid metabolism pathway (CYP301 family) and essential physiological functions (CYP303-305 and CYP15 families), respectively (Fig. 5).

**Fig. 6:**
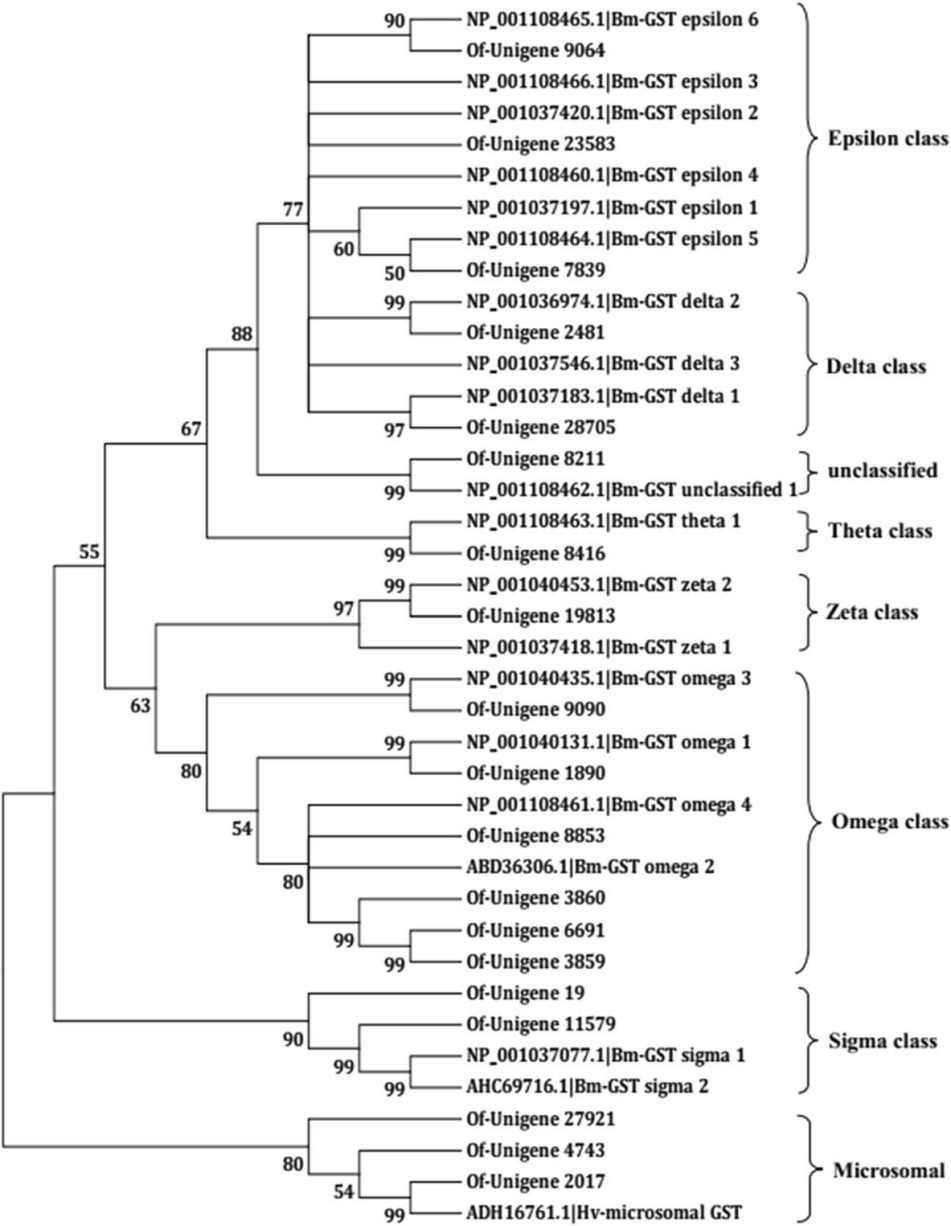
Neighbor-joining phylogenetic analysis of the glutathione S-transferases from *Ostrinia furnacalis* (Of), *Bombyx mori* (Bm) and *Heliothis virescens* (Hv). The tree was constructed from the multiple alignments using MEGA5.0 software and generated with 1000 bootstrap trials using the neighbor-joining method. The numbers indicate the bootstrap confidence values obtained for each node after 1000 repetitions. Positions containing alignment gaps and missing data were eliminated with pairwise deletion

We also identified 63 GST unigenes from the Nr annotation, 19 of which were unique and manually curated (Additional file 4). Based on the closest BLAST hits in the NCBI Nr database and the phylogenetic analysis, these unigenes were assigned to epsilon, delta, theta, zeta, omega, sigma, and microsomal classes (Fig. 6).

**Fig. 7:**
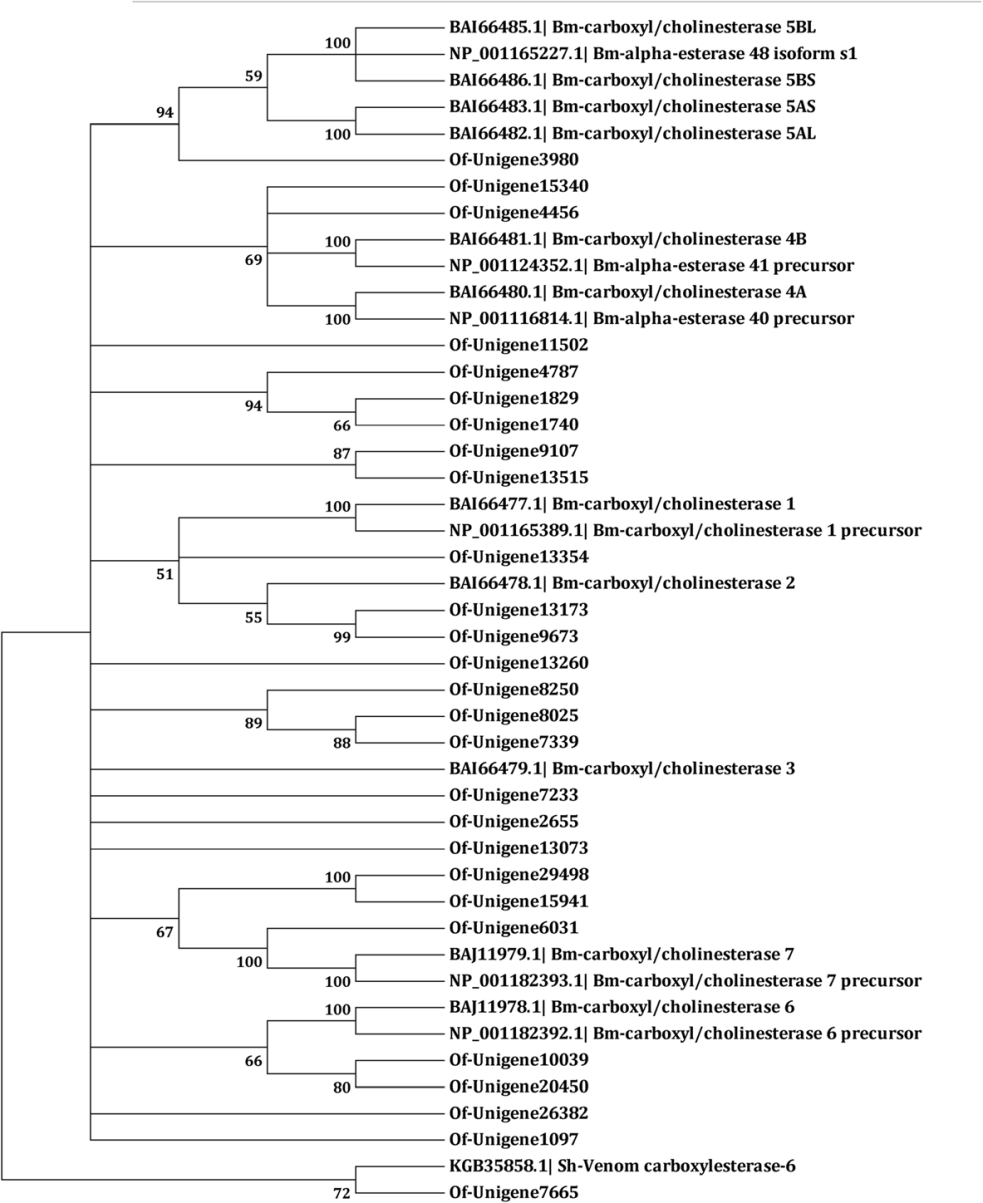
Neighbor-joining phylogenetic analysis of carboxyl/cholinesterases from *Ostrinia furnacalis* (Of), *Bombyx mori* (Bm) and *Schistosoma haematobium* (Sh). The tree was constructed from the multiple alignments using MEGA5.0 software and generated with 1000 bootstrap trials using the neighbor-joining method. The numbers indicate the bootstrap confidence values obtained for each node after 1000 repetitions. Positions containing alignment gaps and missing data were eliminated with pairwise deletion

A total of 171 unigenes with CCE protein motifs were identified. Among these, 27 unigenes were manually curated (Additional file 5), as some of the original sequences were either allelic variants of the same CCE gene or contained short ORFs. Based on phylogenetic analyses with other known CCE genes from other insect species, these 27 sequences were assigned to the known classes of CCEs (Fig. 7).

In addition, a number of unigenes encoding insecticide target proteins, including acetylcholinesterase (AChE), the nicotinic acetylcholine receptor subunits (nAChRs), the gamma-aminobutyric acid (GABA) receptor, the glutamate-gated chloride channel (GluCl), the voltage-gated sodium channel (VGSC) and the ryanodine receptor (RyR), were also identified in the ACB transcriptome (Table 2 and Additional file 6).

**Table 2:** Unigenes related to insecticide target sites in the ACB

Insecticide class	Target site	Gene name	Unigene number	Length (bp)
Organophosphates, Carbamates	Acetylcholinesterase (AChE)	AChE 1	24370	625
			31099	873
		AChE 2	33073	599
Neonicotinoids	Nicotinic acetylcholine receptor (nAChR)	nAChR alpha 1 subunit	18836	1040
		nAChR alpha 4 subunit	30747	148
			20206	399
		nAChR alpha 7 subunit	28176	336
			14229	609
				481
		nAChR alpha 8 subunit	25796	628
		nAChR alpha 9 subunit	8540	1164
		nAChR beta 2 subunit	21446	453
		nAChR beta 3 subunit	33028	372
			6388	1116
Organochlorines, Phenylpyrazoles	GABA receptor	GABA receptor	27882	625
	Glutamate-gated chloride channel (GluCl)	GluCl	19874	812
Pyrethroids, Pyrethrins	Voltage-gated sodium channel (VGSC)	VGSC	25293	580
			33135	391
			33740	734
			22141	614
Diamides (chlorantraniliprole, cyanthraniliprole, flubendiamide)	Ryanodine receptor (RyR)	RyR	6603	4420
			24935	747
			15764	1167
			8793	2358
			25450	234
			6228	2442

### Gene expression profile analysis of responses to flubendiamide

The ACB mRNA samples used for constructing the cDNA library were non-normalized and non-amplified by PCR; thus, the reads in the sequencing dataset most likely represent the relative abundance level of each assembled transcript. To determine whether flubendiamide treatment resulted in statistically significant changes in gene expression, the level of gene expression was determined after normalizing the number of genes in each library to reads per kb per million reads (RPKM). Compared with the control, a total of 1306 genes were affected in the flubendiamide-treated strain, including 415 up-regulated genes and 891 down-regulated genes (Additional file 7, Table 3). More genes were expressed in flubendiamide-treated ACB group (31,478) than in the control (31,059).

**Table 3:** Summary of the differentially expressed unigenes

	Number of unigenes	Percentage %
Total unigenes	34552	
Differentially expressed in the two libraries	1306	3.78
Up (Fl vs. Co)	415	1.20
Down (Fl vs. Co)	891	2.58
Expressed both in Fl and Co	27985	80.99
Expressed only in Fl	3493	10.11
Expressed only in Co	3074	8.90

### Experimental validation

**Fig. 8:**
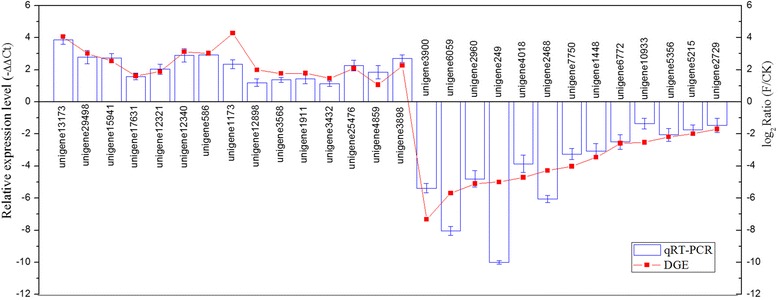
qRT-PCR validation of the differently expressed genes. Fifteen up-regulated genes and thirteen down-regulated genes were identified using qRT-PCR. The 15 up-regulated genes included three esterase genes (unigene13173, unigene29498, and unigene15941), two cytochrome P450 genes (unigene17631 and unigene12321), three hemolin genes (unigene12340, unigene586, and unigene1173), two twitchin genes (unigene12898 and unigene3568), two heat shock protein genes (unigene1911 and unigene3432), two tropomyosin genes (unigene25476 and unigene4859) and one chemosensory protein gene (unigene3898). The 13 down-regulated genes included three cuticular protein genes (unigene3900, unigene4018, and unigene1448), three protease genes (unigene2960, unigene6059, and unigene7750), four oxidoreductase genes (unigene5356, unigene5215, unigene6772, and unigene2729), one neuronal pentraxin gene (unigene249), one glutathione S-transferase gene (unigene10933) and one cytochrome P450 gene (unigene 2468). The left *y*-axis indicates the relative expression levels based on qRT-PCR, and the right *y*-axis indicates the log_2_ ratio of F-treated/Control based on DGE analysis. Error bars indicate the average deviations of the three replicates

To confirm the quality of the transcriptome data and the differential expression results from sequencing and computational analyses, 15 up-regulated genes (three esterase genes: unigene 13173, unigene 29498, unigene 15941; two cytochrome P450 genes: unigene 17631, unigene 12321; three hemolin genes: unigene 12340, unigene 586, unigene 1173; two twitchin genes: unigene 12898, unigene 3568; two heat shock protein 70 genes: unigene 1911, unigene 3432; two tropomyosin genes: unigene 25476, unigene 4859; one chemosensory protein gene: unigene 3898) and 13 down-regulated genes (three cuticular protein genes: unigene 3900, unigene 4018, unigene 1448; three protease genes: unigene 2960, unigene 6059, unigene 7750; four oxidoreductase genes: unigene 5356, unigene 5215, unigene 6772, unigene 2729; one neuronal pentraxin: unigene 249; one glutathione S-transferase: unigene 10933; one cytochrome P450 gene: unigene 2468) were randomly selected for further analysis. These genes were amplified using qRT-PCR, and the qRT-PCR assessments confirmed the direction of change in gene expression based on differential gene expression (DGE) analysis (Fig. 8). Cuticular protein, protease and oxidoreductase had much lower expression levels in response to flubendiamide treatment, while detoxification enzyme (P450s and esterase), muscle contraction-related (twitchin and tropomyosin), immunoglobulin (hemolin), heat shock protein 70 and chemosensory protein genes were significantly overexpressed.

## Discussion

The ACB is the most destructive corn-stalk-boring pest in the countries of East and Southeast Asia, the Pacific and Australasia. In some cases, entire harvests are lost in an outbreak year [13]. However, a lack of genetic information is still a barrier to our knowledge of this insect pest. NGS, developed for rapid sequencing and transcriptomics research, has enabled the use of RNA-Seq and DGE methods to analyze the ACB transcriptome and insecticide mechanisms [14, 15].

For the ACB, as a non-model insect without a reference genome sequence, the clean reads were assembled *de novo* using the short reads assembling programs Mira and CAP3. A total of 35,430 unigenes with a mean length of 716 bp were obtained from the transcriptome analysis. The average length of the unigenes for ACB in the present study was longer than those observed in *Plutella xylostella* (532 bp) [15] and *Liposcelis bostrychophila* (711) [16] but shorter than those in *Blattella germanica* (798 bp) [9] and *Agrotis ipsilon* (770 bp) [17]. The length of the unigenes may be related to the sequencing technique and assembly tools used. BLAST searches against public databases, such as Nt, Nr, Swiss-Prot, GO, COG and KEGG, provided annotation data for the ACB. As expected, the ACB transcripts were homologous to those from more than one species, but most were homologous to Lepidoptera species. The silkworm, *B. mori*, returned the most BLAST hits against the ACB transcripts. This result could reflect the fact that *B. mori* is a Lepidoptera species with a completely sequenced genome [18].

Insecticide detoxification occurs in all insects, and a number of enzymes with insecticide detoxification mechanisms, encoded by members of the P450, GST and CCE multi-gene families, have been identified [19]. Cytochrome P450s are an important enzyme class involved in the metabolism of endogenous substances, such as hormones, fatty acids and steroids, and xenobiotics, such as drugs, pesticides and plant secondary metabolites [20]. The P450 genes represent a large and highly diverse gene family in different species. CYP3 and CYP4 P450 families in insect species have been implicated in the metabolism of plant secondary metabolites and synthetic insecticides [21]. In ACB, the identified cytochrome P450s were assigned to four CYP clans: CYP2, CYP3, CYP4 and the mitochondrial clan; CYP3 was the largest clan. Together, the members of the CYP4, CYP6 and CYP9 families account for 74.2% of the total P450s, indicating that the ACB, similar to other insects, arms itself with a potent capacity for metabolizing various xenobiotics. It has been reported that some P450 genes belonging to the CYP 4, 6, 9 and 12 families are involved in insecticide resistance [16]. For example, the cytochrome P450 gene CYP6BQ23 was recently shown to be the main factor causing pyrethroid resistance in *M. aeneus* across Europe through its hydroxylation of the alcohol moiety of pyrethroids to a less toxic hydroxy metabolite [22]. In *B. tabaci* and *M. persicae*, the cytochrome P450 (CYP6CM1 and CYP6CY3, respectively) over expression contributes to neonicotinoid insecticide resistance because these enzymes can catalyze a more rapid conversion of imidacloprid to its less active form, 5-hydroxy-imidacloprid [23, 24]. Moreover, RNA interference-mediated gene silencing (RNAi) tests revealed that CYP6BG1 overexpression results in permethrin resistance [25]. In the present study, flubendiamide treatment led to up-regulated expression levels of the unigenes 12321, 17631, 11949 and 5498, annotated to CYP337B3, CYP9G3, CYP4C1 and CYP9f2, respectively. CYP337B3 is a chimeric enzyme, capable of metabolizing fenvalerate into non-toxic 4′-hydroxyfenvalerate [26]. Li et al. reported that CYP9G3 plays important roles in the responses to phoxim and cypermethrin metabolism in *Bombyx mandarina* [27]. Methanol exposure significantly up-regulated CYP9f2 mRNA expression; thus, the resulting protein was suggested as a candidate enzyme for methanol metabolism in *Drosophila melanogaster* [28]. In the present study, these overexpressed P450 genes might improve the metabolism and detoxification capacity of the ACB to flubendiamide, and unigenes 12321, 17631, 11949 and 5498 could be involved in the flubendiamide resistance of the ACB.

Known CCEs can be divided into 13 clades; clades A to C are involved in insecticide detoxification [16]. Unigene 13173, localized with mitochondrial, cytosolic and secreted esterases, showed 14.5-fold up-regulation after flubendiamide treatment. This esterase gene might be related to flubendiamide detoxification in the ACB. Unigenes 29498 and 15941, annotated to antennal carboxylesterases, were also dramatically up-regulated. In insects, antennal carboxylesterase plays an important role in the rapid degradation of odorants.

A total of eight classes of GSTs are known; among these, delta and epsilon GSTs are unique to insects and appear to play important roles in xenobiotic detoxification and insecticide resistance [16, 29]. In the ACB transcriptome, five GSTs belonging to the delta and epsilon classes were identified; Hence, it is likely that these five GSTs may have similar roles in flubendiamide detoxification in ACB. Among the other classes of GSTs, such as the omega class, the number of genes identified in the ACB (6) was larger than those in *B. mori*, *L. bostrychophila*, *A. gambiae* and *A. mellifera* [16, 30]. For the sigma and theta classes of GSTs, the numbers of genes (2 and 1, respectively) in the ACB were consistent with those from *B. mori*, and three genes were shown to encode microsomal GSTs, members of which are known in eicosanoid and glutathione metabolism [30, 31].

Five types of genes encoding insecticide target proteins, including the AChE, nAChRs, GABA receptor, GluCl, VGSC and RyR, were identified in the ACB transcriptome. Although many of these genes were not full-length sequences, this information will nevertheless facilitate further characterization of these targets using PCR and/or random amplification of cDNA ends (RACE). Insect AChEs have received much interest, reflecting their role in neurotransmission and because these genes are the principal targets of organophosphate and carbamate insecticides. Three unigenes were identified as containing the *ace* gene in the present study. The nAChRs represent a diverse family of cys-loop ligand-gated ion channels. Ten to twelve nAChR-type receptor gene families have been reported in insects [16]. In the present study, five alpha and two beta subunits were also identified as ACB nAChRs. In contrast, 12 nAChR gene families were identified in *B. mori* [32], while 10 nAChR gene families were identified in *A. gambiae* [33]. The GABA receptors also belong to a superfamily of cys-loop neurotransmitter receptors. Mutation of the GABA-regulated chloride channel can lead to resistance to organochlorine and phenylpyrazole insecticides [34]. Some insect pest populations have evolved modifications of the VGSC protein, which prevent the binding of insecticides (i.e., DDT, pyrethrins and pyrethroids), resulting in the insect developing resistance.

RyRs are members of a superfamily of intracellular Ca^2+^ channels. These channels regulate the release of calcium from the lumen of the sarcoplasmic/endoplasmic reticulum to the cytosol of muscle and non-muscle cells. Insect RyRs are the targets of two classes of diamide chemicals [8, 35]. The homomeric tetramer RyR is the largest known ion channel, with each monomer consisting of approximately 5000 amino acids. A total of six unigenes were identified as part of the *O. furnacalis* RyR (*OfRyR*) gene, with the longest unigene being 4420 bp. In the present study, the *OfRyR* gene was up-regulated after flubendiamide exposure compared with the control, but this difference was not statistically significant. Actually, the gene expression of RyR changed depending on insect’s life cycle. The expression level of RyR differed among different developmental stages. For example, the *Of*RyR showed the lowest expression level in egg and the highest in adult [8]. Meanwhile, the *Px*RyR was highly expressed in the second-instar larvae and adults, and their relative expression levels were 12.6 and 11.8 times than that in the prepupae, which showed the lowest expression level of *Px*RyR [36]. Furthermore, the genes involved in calcium signaling and muscle control pathways, such as the twitchin genes (unigenes 4859 and 12898), were also significantly overexpressed after flubendiamide treatment. These results suggested that pathways involving multiple genes, such as calcium signaling, muscle control and metabolic pathways, play dominant roles in flubendiamide exposure.

Studying the temporal transcriptome responses of insects to xenobiotics could lead to the discovery of novel molecular mechanisms contributing to insecticide detoxification and tolerance. Gene expression analysis after flubendiamide exposure will significantly complement and enrich the ACB gene expression data and facilitate the discovery of novel genes, gene functions and insecticidal targets of the ACB. In insects, the response to environmental stress triggers the expression of various proteins, including heat shock proteins, metallothioneins or p-glycoprotein [20]. The data obtained in the present study revealed that the transcription levels of heat shock protein 70 genes were significantly up-regulated in response to flubendiamide exposure. However, some genes encoding enzymes involved in the production of cellular catabolism, such as trypsin and serine protease, were down-regulated in the ACB in response to flubendiamide, and the cuticular protein genes also showed much lower expression levels in flubendiamide treatment. The down-regulation of cuticular-related proteins indicated that flubendiamide might play a role in thinning the cuticles in the ACB. Indeed, thickened cuticles affect the rates of insecticide penetration [37]. Thus, thinned cuticles will accelerate the transportation of flubendiamide to the target and improve its insecticidal efficacy.

## Conclusions

In conclusion, the present study represents a functional transcriptome analysis of the ACB. The differential gene expression data elucidated the molecular mechanisms of the ACB in response to the novel diamide insecticide flubendiamide. In addition, this investigation will facilitate identification of the genes involved in insecticide resistance and may assist in designing new compounds or other strategies for the control of the ACB.

## Methods

### Sample preparation

The ACB laboratory colony was originally obtained from a stalk of field corn at the Hengshui experimental field of the Chinese Academy of Agricultural Sciences, Hebei Province, China. The colony was maintained on an agar-free semi-artificial diet in the laboratory at 26 ± 1 °C, with 70% relative humidity and a 16:8 (L:D) photoperiod. ACBs for the generation of cDNA libraries were obtained from two different strains. One strain was used as a control strain and was fed on a semi-artificial diet without insecticide treatment, and the other strain was exposed to a diet containing flubendiamide at the 95% lethal concentration value (3.8 μg g^−1^). After 48 h, more than 50 surviving 2nd instar larvae of each strain were collected in two separate tubes. The ACB samples were immediately snap-frozen in liquid N_2_ and stored at −80 °C until further use.

### RNA extraction, cDNA library construction, and PGM sequencing

Total RNA from each sample (flubendiamide-treated and control ACBs) was isolated using the RNeasy^®^ Mini Kit (Qiagen, Germany), according to the manufacturer’s instructions. The integrity of the total RNA was confirmed using both 1.5% agarose gel electrophoresis and an Agilent 2100 Bioanalyzer (Palo Alto, USA). The quantity of the total RNA was determined using a NanoDrop 1000 spectrophotometer (Thermo, USA). Additionally, mRNA samples were isolated from each total RNA using PolyATtract^®^ mRNA isolation systems (Promega, USA). According to the manufacturer’s instructions, the mRNA was purified using polyT oligo-attached magnetic beads. The mRNA was broken into short fragments (200–800 nucleotides) in the presence of fragmentation buffer at 70 °C for 1 min. These short fragments were used as templates for first-strand cDNA synthesis with random primers. Subsequently, second-strand cDNAs were synthesized using a reaction system containing buffer, dNTPs, RNaseH and DNA polymerase I. These cDNA fragments were purified using a QIAquick Gel Extraction Kit (Qiagen, Germany) and were subjected to end repair by the addition of an A base to the 3′ end. Subsequently, the cDNAs were connected with sequencing adapters. Suitable fragments (400–1000 bp) were extracted and amplified by emulsion PCR (emPCR) to construct a cDNA library. The cDNA libraries were sequenced on a PGM platform using the Ion PGM ™ sequencing 400 Kit (Life Technologies, USA).

### *De novo* transcriptome assembly and analysis

The raw reads were produced by base calling, which transforms pyroluminescence intensity signals to nucleotide sequences. Raw reads were preprocessed using the in-house developed programs Dust [38], FastqMcf (http://code.google.com/p/ea-utils/wiki/FastqMcf) and seq_crumbs (http://bioinf.comav.upv.es/seq_crumbs) to trim the adapters and poly A/T tails and to remove short (<100 bp), low-quality contaminating sequences. The resulting clean reads from both treatments were assembled into unigene sequences using the Mira [39] and CAP3 (http://seq.cs.iastate.edu) assembler programs at default parameters.

All *de novo* assembled unigenes were BLASTx searched against the Nr protein and Swiss-Prot databases. The sequences with no BLASTx hits were searched against the NCBI Nt database using BLASTn [16]. The best matches were used to identify coding regions and to determine the sequence direction. GO, COG and KEGG analyses were used for the functional classification of the annotated unigenes. For the GO analysis, both BLAST2GO and WEGO were employed. These programs were used to extract the GO terms associated with homologies identified with BLAST, returning a list of GO annotations represented as hierarchical categories of increasing specificity [40]. COG and KEGG annotations were analyzed using the Blastall program against the COG (http://www.ncbi.nlm.gov/COG/) and KEGG (http://www.genome.jp/kegg/) databases [41]. Potential ORFs were identified using the ORF finder (http://www.ncbi.nlm.nih.gov/gorf/gorf.html). Simple sequence repeats were identified using MISA (http://pgrc.ipk-gatersleben.de/misa/) [42].

### Analysis of genes related to insecticide detoxification and target proteins

Sequences encoding insecticide target proteins, such as AChE, nAChRs, GABA, GluCl, VGSC and RyR were identified from the Nr database with an *E*-value cut-off of 10^−5^. The genes related to insecticide-detoxification enzymes (P450s, CCEs and GSTs) were also manually curated using the above methods. All of the confirmed protein sequences of P450s, CCEs and GSTs were used for alignment and phylogenetic analysis. MEGA5.0 software was used to perform multiple sequence alignment prior to phylogenetic analysis and to construct consensus phylogenetic trees using the neighbor-joining method with p-distance under default parameters. Bootstrap analysis of 1000 replications was performed to evaluate the branch strength of each tree.

### Differentially expressed unigene detection

The gene expression level was calculated using the RPKM method [43]. The criteria of *P* < 0.01 and the absolute value of log_2_ ratio ≥ 1 were used to judge the significance of differences in gene expression between the two samples (flubendiamide-treated ACB and control). More stringent criteria, with smaller P and larger fold-change values, were used to identify differentially expressed genes [44].

### Experimental validation

qRT-PCR was performed to confirm the differently expressed genes. Total RNA from two samples (flubendiamide-treated and control ACBs) was extracted as described above. Complementary DNA was synthesized using an equal amount of total RNA (1 μg) from the two samples according to the instructions of the PrimeScript™ RT Reagent Kit with gDNA Eraser (Perfect Real Time) (Takara, Dalian, China). The primers employed in qRT-PCR are listed in Additional file 8. Each 20 μL of PCR master mix contained 2 μL of diluted cDNA template, 10 μL of SYBR Premix Ex Taq™, 0.4 μL of forward primer (10 μM), 0.4 μL of reverse primer (10 μM), 0.4 μL of Rox Reference Dye II (50×) and 6.8 μL of RNase-free H_2_O, according to the instructions of SYBR^®^ Premix Ex Taq™ (Perfect Real Time) (Takara, Dalian, China). Thermal cycling was completed on a 7500 Real-time PCR system (Applied Biosystems, USA) using the following program: 95 °C for 30 s, followed by 40 cycles of 95 °C for 5 s and 60 °C for 34 s. Subsequently, a melting curve analysis from 60 °C to 90 °C was applied to all reactions to verify a single PCR product. After which the average cycle threshold (Ct) was calculated per sample. The housekeeping gene *β-actin* from the ACB was used as a previously evaluated internal control and was applied as an appropriate reference for insecticide-induced gene expression profiling [45]. The fold change of target genes was calculated using the relative quantitative method (2^-△△Ct^) [46]. To check reproducibility, four technical replicates and three biological replicates were carried out for each treatment. Statistical analysis was performed using Duncan’s Multiple Range Test for significance (*P* < 0.05) in SPSS 17.0 (SPSS, Inc., Chicago, IL, USA).

## Additional files

Additional file 1: GO classifications of the ACB unigenes. (DOCX 15 kb)
Additional file 2: KEGG pathway annotation of the ACB unigenes. (DOCX 25 kb)
Additional file 3: Cytochrome P450 nucleotide sequences of the ACB. (TXT 48 kb)
Additional file 4: Glutathione S-transferase nucleotide sequences of the ACB. (TXT 17 kb)
Additional file 5: Carboxyl/cholinesterase nucleotide sequences of the ACB. (TXT 43 kb)
Additional file 6: Nucleotide sequences of the target sites of the most important insecticide classes. (TXT 25 kb)
Additional file 7: Differentially expressed genes between flubendiamide-treated and control ACBs. (XLS 27928 kb)
Additional file 8: Information for the primers used in the qRT-PCR analysis. (DOCX 16 kb)

## Abbreviations

ACB: Asian corn borer
AChE: Acetylcholinesterase
CCE: Carboxyl/cholinesterase
COG: Clusters of orthologous groups
CYP: Cytochrome P450 monooxygenase
EST: Expressed sequenced tag
GABA: Gamma-aminobutyric acid receptor
GluCl: Glutamate-gated chloride channel
GO: Gene ontology
GST: Glutathione S-transferase
KEGG: Kyoto Encyclopedia of Genes and Genomes
nAChR: nicotinic acetylcholine receptor
NGS: Next-generation sequencing
Nr: Non-redundant protein database
ORF: Open reading frame
PCR: Polymerase chain reaction
PGM: Personal genome machine
RyR: Ryanodine receptor
VGSC: Voltage-gated sodium channel

## References

[1] He KL, Wang ZY, Bai SX, Zheng L, Wang YB, Cui HY (2006). Efficacy of transgenic Bt cotton for resistance to the Asian corn borer (Lepidoptera: Crambidae). Crop Prot.

[2] Jin TT, Chang X, Gatehouse AMR, Wang ZY, Edwards MG, He KL (2014). Downregulation and mutation of a cadherin gene associated with Cry1Ac resistance in the Asian corn borer, *Ostrinia furnacalis* (Guenée). Toxins.

[3] Yang DB, Zhang LN, Yan XJ, Wang ZY, Yuan HZ (2014). Effects of droplet distribution on insecticide toxicity to Asian corn borers (*Ostrinia furnaealis*) and spiders (*Xysticus ephippiatus*). J Integr Agric.

[4] Folcher L, Jarry M, Weissenberger A, Gérault F, Eychenne N, Delos M, Regnault-Roger C (2009). Comparative activity of agrochemical treatments on mycotoxin levels with regard to corn borers and *Fusarium mycoflora* in maize (*Zea mays L.*) fields. Crop Prot.

[5] Kato K, Kiyonaka S, Sawaguchi Y, Tohnishi M, Masaki T, Yasokawa N, Mizuno Y, Mori E, Inoue K, Hamachi I, Takeshima H, Mori Y (2009). Molecular characterization of flubendiamide sensitivity in lepidopterous ryanodine receptor Ca^2+^ release channel. Biochemistry.

[6] Ebbinghaus-Kintscher U, Luemmen P, Lobitz N, Schulte T, Funke C, Fischer R, Masaki T, Yasokawa N, Tohnishi M (2006). Phthalic acid diamides activate ryanodine sensitive Ca^2+^ release channels in insects. Cell Calcium.

[7] Cordova D, Benner EA, Sacher MD, Rauh JJ, Sopa JS, Lahm GP, Selby TP, Stevenson TM, Flexner L, Gutteridge S, Rhoades DF, Wu L, Smith RM, Tao Y (2006). Anthranilic diamides: A new class of insecticides with a novel mode of action, ryanodine receptor activation. Pestic Biochem Physiol.

[8] Cui L, Yang DB, Yan XJ, Rui CH, Wang ZY, Yuan HZ (2013). Molecular cloning, characterization and expression profiling of a ryanodine receptor gene in Asian corn borer, *Ostrinia furnacalis* (Guenée). PLoS ONE.

[9] Zhou XJ, Qian K, Tong Y, Zhu JJ, Qiu XH, Zeng XP (2014). De novo transcriptome of the hemimetabolous German cockroach (*Blattella germanica*). PLoS ONE.

[10] Feyereisen R, Gilbert LI (2012). Insect CYP genes and P450 enzymes. Insect Molecular Biology and Biochemistry.

[11] Harris MA, Clark J, Ireland A, Lomax J, Ashburner M, Foulger R, Eilbeck K, Lewis S, Marshall B, Mungall C, Richter J, Rubin GM, Blake JA, Bult C, Dolan M, Drabkin H, Eppig JT, Hill DP, Ni L, Ringwald M, Balakrishnan R, Cherry JM, Christie KR, Costanzo MC, Dwight SS, Engel S, Fisk DG, Hirschman JE, Hong EL, Nash RS (2004). The Gene Ontology (GO) database and informatics resource. Nucleic Acids Res.

[12] Tang LD, Wang XM, Jin FL, Qiu BL, Wu JH, Ren SX (2014). De novo sequencing-based transcriptome and digital gene expression analysis reveals insecticide resistance-relevant genes in *Propylaea japonica* (Thunberg) (Coleoptea: Coccinellidae). PLoS One.

[13] Xu LN, Ling YH, Wang YQ, Wang ZY, Hu BJ, Zhou ZY, Hu F, He KL (2016). Identification of differentially expressed microRNAs between *Bacillus thuringiensis* Cry1Ab resistant and -susceptible strains of *Ostrinia furnacalis*. Sci Rep.

[14] Xu Z, Zhu W, Liu Y, Liu X, Chen Q, Peng M, Wang XZ, Shen GM, He L (2014). Analysis of insecticide resistance-related genes of the carmine spider mite *Tetranychus cinnabarinus* based on a de novo assembled transcriptome. PLoS One.

[15] Lin Q, Jin F, Hu Z, Chen H, Yin F, Li Z, Dong XL, Zhang DY, Ren SX, Feng X (2013). Transcriptome analysis of chlorantraniliprole resistance development in the Diamondback moth *Plutella xylostella*. PLoS One.

[16] Dou W, Shen GM, Niu JZ, Ding TB, Wei DD, Wang JJ (2013). Mining genes involved in insecticide resistance of *Liposcelis bostrychophila* Badonnel by transcriptome and expression profile analysis. PLoS ONE.

[17] Gu SH, Wu KM, Guo YY, Pickett JA, Field LM, Zhou JJ, Zhang YJ (2013). Identification of genes expressed in the sex pheromone gland of the black cutworm *Agrotis ipsilon* with putative roles in sex pheromone biosynthesis and transport. BMC Genomics.

[18] Xia QY, Zhou ZY, Lu C, Cheng DJ, Dai FY, Li B, Zhao P, Zha XF, Cheng TC, Chai CL, Pan GQ, Xu JS, Liu C, Lin Y, Qian JF, Hou Y, Wu ZL, Li GR, Pan MH, Li CF, Shen YH, Lan XQ, Yuan LW, Li T, Xu HF, Yang GW, Wan YJ, Zhu Y, Yu MD, Shen WD (2004). A draft sequence for the genome of the domesticated silkworm (*Bombyx mori*). Science.

[19] Li X, Schuler MA, Berenbaum MR (2007). Molecular mechanisms of metabolic resistance to synthetic and natural xenobiotics. Annu Rev Entomol.

[20] Zimmer CT, Maiwald F, Schorn C, Bass C, Ott M, Nauen R (2014). A de novo transcriptome of European pollen beetle populations and its analysis, with special reference to insecticide action and resistance. Insect Mol Biol.

[21] Karatolos N, Pauchet Y, Wilkinson P, Chauhan R, Denholm I, Gorman K, Nelson DR, Bass C, Hffrench-Constant R, Williamson MS (2011). Pyrosequencing the transcriptome of the greenhouse whitefly, *Trialeurodes vaporariorum* reveals multiple transcripts encoding insecticide targets and detoxifying enzymes. BMC Genomics.

[22] Zimmer CT, Bass C, Williamson MS, Kaussmann M, Wölfel K, Gutbrod O, Nauena R (2014). Molecular and functional characterization of CYP6BQ23, a cytochrome P450 conferring resistance to pyrethroids in European populations of pollen beetle, *Meligethes aeneus*. Insect Biochem Mol Biol.

[23] Karunker I, Morou E, Nikou D, Nauen R, Sertchook R, Stevenson BJ (2009). Structural model and functional characterization of the *Bemisia tabaci* CYP6CM1vQ, a cytochrome P450 associated with high levels of imidacloprid resistance. Insect Biochem Mol Biol.

[24] Puinean AM, Foster SP, Oliphant L, Denholm I, Field LM, Millar NS, Williamson MS, Bass C (2010). Amplification of a cytochrome P450 gene is associated with resistance to neonicotinoid insecticides in the aphid *Myzus persicae*. PLoS Genet.

[25] Bautista MAM, Miyata T, Miura K, Tanaka T (2009). RNA interference-mediated knockdown of a cytochrome P450, CYP6BG1, from the diamond back moth, *Plutella xylostella*, reduces larval resistance to permethrin. Insect Biochem Mol Biol.

[26] Joußen N, Agnolet S, Lorenz S, Schöne SE, Ellinger R, Schneider B, Heckela DG (2012). Resistance of Australian *Helicoverpa armigera* to fenvalerate is due to the chimeric P450 enzyme CYP337B3. Proc Natl Acad Sci U S A.

[27] Li F, Ni M, Zhang H, Wang BB, Xu KZ, Tian JH, Hu JS, Shen WD, Li B (2015). Expression profile analysis of silkworm P450 family genes after phoxim induction. Pestic Biochem Physiol.

[28] Wang SP, He GL, Chen RR, Li F, Li GQ (2012). The involvement of cytochrome P450 monooxygenases in methanol elimination in *Drosophila melanogaster* larvae. Arch Insect Biochem Physiol.

[29] Ranson H, Claudianos C, Ortelli F, Abgrall C, Hemingway J, Sharakhova MV, Unger MF, Collins FH, Feyereisen R (2002). Evolution of supergene families associated with insecticide resistance. Science.

[30] Claudianos C, Ranson H, Johnson RM, Biswas S, Schuler MA, Berenbaum MR, Feyereisen R, Oakeshott JG (2006). A deficit of detoxification enzymes: pesticide sensitivity and environmental response in the honeybee. Insect Mol Biol.

[31] Sheehan D, Meade G, Foley VM, Dowd CA (2001). Structure, function and evolution of glutathione transferases: implications for classification of non-mammalian members of an ancient enzyme superfamily. Biochem J.

[32] Shao YM, Dong K, Zhang CX (2007). The nicotinic acetylcholine receptor gene family of the silkworm, *Bombyx mori*. BMC Genomics.

[33] Jones AK, Grauso M, Sattelle DB (2005). The nicotinic acetylcholine receptor gene family of the malaria mosquito, *Anopheles gambiae*. Genomics.

[34] Hosie AM, Baylis HA, Buckingham SD, Sattelle DB (1995). Actions of the insecticide fipronil, on dieldrin-sensitive and -resistant GAGB receptors of *Drosophila melanogaster*. Br J Pharmacol.

[35] Sun LN, Qiu GS, Cui L, Ma CS, Yuan HZ (2015). Molecular characterization of a ryanodine receptor gene from *Spodoptera exigua* and its upregulation by chlorantraniliprole. Pestic Biochem Physiol.

[36] Guo L, Tang BZ, Dong W, Liang P, Gao XW (2012). Cloning, characterisation and expression profiling of the cDNA encoding the ryanodine receptor in diamondback moth, *Plutella xylostella* (L.) (Lepidoptera: Plutellidae). Pest Manag Sci.

[37] Wood O, Hanrahan S, Coetzee M, Koekemoer L, Brooke B (2010). Cuticle thickening associated with pyrethroid resistance in the major malaria vector *Anopheles funestus*. Parasit Vectors.

[38] Morgμlis A, Gertz EM, Schaffer AA, Agarwala R (2006). A fast and symmetric DUST implementation to mask low-complexity DNA sequences. J Comput Biol.

[39] Chevreux B, Pfisterer T, Drescher B, Driesel AJ, Muller WE, Wetter T, Suhai S (2004). Using the miraEST assembler for reliable and automated mRNA transcript assembly and SNP detection in sequenced ESTs. Genome Res.

[40] Ashburner M, Ball CA, Blake JA, Botstein D, Butler H, Cherry JM, Davis AP, Dolinski K, Dwight SS, Eppig JT (2000). Gene Ontology: tool for the unification of biology. Nat Genet.

[41] Salomonis N, Hanspers K, Zambon AC, Vranizan K, Lawlor SC, Dahlquist KD, Doniger SW, Stuart J, Conklin BR, Pico AR (2007). GenMAPP 2: new features and resources for pathway analysis. BMC Bioinformatics.

[42] Thiel T, Michalek W, Varshney RK, Graner A (2003). Exploiting EST databases for the development and characterization of gene-derived SSR-markers in barley (*Hordeum vμlgare* L.). Theor Appl Genet.

[43] Mortazavi A, Williams BA, Mccue K, Schaeffer L, Wold B (2008). Mapping and quantifying mammalian transcriptomes by RNA-Seq. Nat Methods.

[44] Audic S, Claverie JM (1997). The significance of digital gene expression profiles. Genome Res.

[45] Grabherr MG, Haas BJ, Yassour M, Levin JZ, Thompson DA, Amit I, Adiconis X, Fan L, Raychowdhury R, Zeng QD, Chen ZH, Mauceli E, Hacohen N, Gnirke A, Rhind N, Palma F, Birren BW, Nusbaum C, Lindblad-Toh K, Friedman N, Regev A (2011). Full length transcriptome assembly from RNA-Seq data without a reference genome. Nat Biotechnol.

[46] Pfaffl MW (2001). A new mathematical model for relative quantification in real-time RT-PCR. Nucleic Acids Res.

