# Supplementary material for: De novo transcriptome and expression profile analyses of the Asian corn borer (Ostrinia furnacalis) reveals relevant flubendiamide response genes
Source: BMC Genomics. 2017 Jan 5;18:20. doi: 10.1186/s12864-016-3431-6 (PMC5217215; doi:10.1186/s12864-016-3431-6)
Supplement: Additional file 1: — GO classifications of the ACB unigenes. (DOCX 15 kb) [file 12864_2016_3431_MOESM1_ESM.docx]

**Table S1 GO classifications of *Ostrinia furnacalis* unigenes**

| **GO** | **Class** | **No. of unigenes from control ACB** | **No. of unigenes from flubendiamide-treated ACB** |
| --- | --- | --- | --- |
| **Cellular Component** | cell | 6064 | 6064 |
|  | cell part | 5679 | 5672 |
|  | extracellular region | 107 | 108 |
|  | extracellular region part | 65 | 65 |
|  | macromolecular | 1542 | 1545 |
|  | membrane-enclosed | 420 | 415 |
|  | organelle | 3622 | 3601 |
|  | organelle part | 1820 | 1805 |
|  | synapse | 7 | 7 |
|  | synapse part | 3 | 3 |
|  | virion | 3 | 3 |
| **Molecular Function** | antioxidant activity | 23 | 22 |
|  | binding | 4377 | 4418 |
|  | catalytic activity | 5024 | 5058 |
|  | channel regulator activity | 1 | 1 |
|  | enzyme regulator activity | 198 | 202 |
|  | molecular transducer activity | 334 | 345 |
|  | nucleic acid binding transcription factor activity | 21 | 20 |
|  | protein binding transcription factor activity | 66 | 67 |
|  | structural molecule activity | 21 | 19 |
|  | transcription regulator activity | 120 | 126 |
|  | translation regulator activity | 8 | 8 |
|  | transporter activity | 629 | 635 |
| **Biological Process** | biological adhesion | 112 | 118 |
|  | biological regulation | 1915 | 1952 |
|  | cell killing | 2 | 2 |
|  | cell proliferation | 54 | 54 |
|  | cellular component organization or biogenesis | 1837 | 1836 |
|  | cellular process | 5567 | 5609 |
|  | death | 315 | 333 |
|  | developmental process | 1944 | 1969 |
|  | establishment of localization | 1525 | 1521 |
|  | growth | 215 | 226 |
|  | immune system process | 179 | 184 |
|  | localization | 1837 | 1836 |
|  | locomotion | 347 | 349 |
|  | metabolic process | 4898 | 4911 |
|  | multi-organism process | 174 | 178 |
|  | multicellular organismal process | 1917 | 1939 |
|  | negative regulation of biological process | 384 | 395 |
|  | pigmentation | 46 | 48 |
|  | positive regulation of biological process | 221 | 227 |
|  | regulation of biological process | 1574 | 1605 |
|  | reproduction | 571 | 578 |
|  | reproductive process | 565 | 572 |
|  | response to stimulus | 1246 | 1246 |
|  | rhythmic process | 29 | 29 |
|  | signaling | 1138 | 1154 |
|  | signaling process | 706 | 721 |
|  | viral reproduction | 19 | 20 |
