# Supplementary material for: De novo transcriptome and expression profile analyses of the Asian corn borer (Ostrinia furnacalis) reveals relevant flubendiamide response genes
Source: BMC Genomics. 2017 Jan 5;18:20. doi: 10.1186/s12864-016-3431-6 (PMC5217215; doi:10.1186/s12864-016-3431-6)
Supplement: Additional file 2: — KEGG pathway annotation of the ACB unigenes. (DOCX 25 kb) [file 12864_2016_3431_MOESM2_ESM.docx]

**Table S2 KEGG pathway annotation of *Ostrinia furnacalis* unigenes**

| **Code** | **Pathway** | **No. of unigenes from control ACB** | **No. of unigenes from flubendiamide-treated ACB** |
| --- | --- | --- | --- |
| 1 | Metabolic pathways | 2722 | 2711 |
| 2 | Biosynthesis of secondary metabolites | 971 | 970 |
| 3 | Purine metabolism | 393 | 398 |
| 4 | Focal adhesion | 322 | 329 |
| 5 | Phagosome | 318 | 310 |
| 6 | Spliceosome | 300 | 298 |
| 7 | Protein processing in endoplasmic reticulum | 300 | 300 |
| 8 | Ribosome | 290 | 290 |
| 9 | Lysosome | 265 | 264 |
| 10 | Oxidative phosphorylation | 252 | 250 |
| 11 | Aminobenzoate degradation | 246 | 255 |
| 12 | Endocytosis | 237 | 236 |
| 13 | Peroxisome | 237 | 232 |
| 14 | Glycerolipid metabolism | 230 | 218 |
| 15 | Metabolism of xenobiotics by cytochrome P450 | 226 | 222 |
| 16 | Ubiquitin mediated proteolysis | 224 | 220 |
| 17 | Tight junction | 217 | 226 |
| 18 | Protein digestion and absorption | 210 | 211 |
| 19 | Retinol metabolism | 207 | 206 |
| 20 | Pyrimidine metabolism | 204 | 203 |
| 21 | RNA transport | 203 | 201 |
| 22 | MAPK signaling pathway | 198 | 215 |
| 23 | Pyruvate metabolism | 189 | 185 |
| 24 | Calcium signaling pathway | 185 | 196 |
| 25 | Tyrosine metabolism | 185 | 192 |
| 26 | Steroid hormone biosynthesis | 184 | 182 |
| 27 | Starch and sucrose metabolism | 180 | 184 |
| 28 | Cell cycle | 168 | 164 |
| 29 | Glycolysis/Gluconeogenesis | 163 | 162 |
| 30 | Amino sugar and nucleotide sugar metabolism | 153 | 150 |
| 31 | Pentose and glucuronate interconversions | 148 | 142 |
| 32 | Bisphenol degradation | 148 | 148 |
| 33 | Citrate cycle (TCA cycle) | 147 | 144 |
| 34 | Linoleic acid metabolism | 146 | 147 |
| 35 | Limonene and pinene degradation | 144 | 149 |
| 36 | Wnt signaling pathway | 144 | 145 |
| 37 | RNA degradation | 143 | 141 |
| 38 | Adherens junction | 141 | 137 |
| 39 | Glycine, serine and threonine metabolism | 136 | 131 |
| 40 | Glutathione metabolism | 132 | 128 |
| 41 | Galactose metabolism | 131 | 135 |
| 42 | Arginine and proline metabolism | 129 | 129 |
| 43 | Fructose and mannose metabolism | 125 | 123 |
| 44 | Valine, leucine and isoleucine degradation | 124 | 121 |
| 45 | Fatty acid metabolism | 121 | 116 |
| 46 | Proteasome | 119 | 124 |
| 47 | Gap junction | 115 | 115 |
| 48 | Inositol phosphate metabolism | 114 | 118 |
| 49 | Tropane, piperidine and pyridine alkaloid biosynthesis | 109 | 111 |
| 50 | Glycerophospholipid metabolism | 108 | 106 |
| 51 | Polycyclic aromatic hydrocarbon degradation | 106 | 109 |
| 52 | Propanoate metabolism | 106 | 100 |
| 53 | Ascorbate and aldarate metabolism | 104 | 102 |
| 54 | Aminoacyl-tRNA biosynthesis | 104 | 108 |
| 55 | Phosphatidylinositol signaling system | 103 | 109 |
| 56 | Lysine degradation | 102 | 99 |
| 57 | Pentose phosphate pathway | 101 | 96 |
| 58 | Phenylalanine metabolism | 100 | 98 |
| 59 | Complement and coagulation cascades | 100 | 95 |
| 60 | Fat digestion and absorption | 97 | 94 |
| 61 | Tryptophan metabolism | 95 | 95 |
| 62 | Carbon fixation in photosynthetic organisms | 92 | 92 |
| 63 | ABC transporters | 92 | 95 |
| 64 | Porphyrin and chlorophyll metabolism | 90 | 86 |
| 65 | Adipocytokine signaling pathway | 90 | 90 |
| 66 | Methane metabolism | 88 | 87 |
| 67 | Biosynthesis of unsaturated fatty acids | 88 | 84 |
| 68 | Alanine, aspartate and glutamate metabolism | 87 | 87 |
| 69 | Vasopressin-regulated water reabsorption | 85 | 89 |
| 70 | RNA polymerase | 84 | 84 |
| 71 | mRNA surveillance pathway | 81 | 83 |
| 72 | GnRH signaling pathway | 80 | 85 |
| 73 | Stilbenoid, diarylheptanoid and gingerol biosynthesis | 80 | 83 |
| 74 | Other types of O-glycan biosynthesis | 80 | 81 |
| 75 | ErbB signaling pathway | 75 | 81 |
| 76 | Ubiquinone and other terpenoid-quinone biosynthesis | 73 | 73 |
| 77 | Cell adhesion molecules (CAMs) | 73 | 75 |
| 78 | Phenylpropanoid biosynthesis | 73 | 74 |
| 79 | Mineral absorption | 71 | 76 |
| 80 | Long-term potentiation | 69 | 73 |
| 81 | Butanoate metabolism | 69 | 67 |
| 82 | Notch signaling pathway | 69 | 68 |
| 83 | Nucleotide excision repair | 69 | 63 |
| 84 | beta-Alanine metabolism | 68 | 68 |
| 85 | Basal transcription factors | 68 | 69 |
| 86 | One carbon pool by folate | 66 | 67 |
| 87 | Chloroalkane and chloroalkene degradation | 65 | 64 |
| 88 | Carbohydrate digestion and absorption | 63 | 73 |
| 89 | Toll-like receptor signaling pathway | 62 | 66 |
| 90 | Arachidonic acid metabolism | 61 | 61 |
| 91 | Plant-pathogen interaction | 61 | 59 |
| 92 | Fatty acid biosynthesis | 60 | 59 |
| 93 | DNA replication | 59 | 52 |
| 94 | Proximal tubule bicarbonate reclamation | 58 | 57 |
| 95 | Histidine metabolism | 58 | 58 |
| 96 | Cyanoamino acid metabolism | 58 | 57 |
| 97 | Riboflavin metabolism | 57 | 61 |
| 98 | alpha-Linolenic acid metabolism | 55 | 55 |
| 99 | Isoquinoline alkaloid biosynthesis | 54 | 56 |
| 100 | TGF-beta signaling pathway | 52 | 55 |
| 101 | N-Glycan biosynthesis | 52 | 50 |
| 102 | Cysteine and methionine metabolism | 50 | 48 |
| 103 | Valine, leucine and isoleucine biosynthesis | 49 | 48 |
| 104 | Long-term depression | 48 | 54 |
| 105 | Apoptosis | 48 | 53 |
| 106 | Vitamin digestion and absorption | 48 | 46 |
| 107 | Protein export | 48 | 40 |
| 108 | Glyoxylate and dicarboxylate metabolism | 47 | 48 |
| 109 | Terpenoid backbone biosynthesis | 47 | 45 |
| 110 | Natural killer cell mediated cytotoxicity | 47 | 46 |
| 111 | Endocrine and other factor-regulated calcium reabsorption | 46 | 49 |
| 112 | Benzoate degradation | 46 | 48 |
| 113 | Fc epsilon RI signaling pathway | 46 | 51 |
| 114 | Sphingolipid metabolism | 46 | 44 |
| 115 | Base excision repair | 46 | 43 |
| 116 | Naphthalene degradation | 45 | 47 |
| 117 | Ether lipid metabolism | 44 | 47 |
| 118 | Ribosome biogenesis in eukaryotes | 44 | 46 |
| 119 | Caprolactam degradation | 43 | 42 |
| 120 | Nitrogen metabolism | 43 | 44 |
| 121 | Steroid biosynthesis | 40 | 39 |
| 122 | Olfactory transduction | 39 | 40 |
| 123 | Nicotinate and nicotinamide metabolism | 39 | 38 |
| 124 | Other glycan degradation | 39 | 36 |
| 125 | Pantothenate and CoA biosynthesis | 37 | 36 |
| 126 | Primary bile acid biosynthesis | 37 | 35 |
| 127 | Glycosylphosphatidylinositol (GPI)-anchor biosynthesis | 37 | 39 |
| 128 | Betalain biosynthesis | 36 | 39 |
| 129 | Cytosolic DNA-sensing pathway | 36 | 32 |
| 130 | NOD-like receptor signaling pathway | 35 | 37 |
| 131 | Styrene degradation | 35 | 37 |
| 132 | Insect hormone biosynthesis | 34 | 34 |
| 133 | Various types of N-glycan biosynthesis | 34 | 35 |
| 134 | Ethylbenzene degradation | 33 | 35 |
| 135 | Glycosaminoglycan degradation | 33 | 32 |
| 136 | Selenocompound metabolism | 32 | 32 |
| 137 | Cytokine-cytokine receptor interaction | 32 | 35 |
| 138 | Phototransduction | 31 | 30 |
| 139 | Sulfur metabolism | 31 | 29 |
| 140 | Homologous recombination | 30 | 32 |
| 141 | Folate biosynthesis | 29 | 30 |
| 142 | SNARE interactions in vesicular transport | 29 | 29 |
| 143 | Caffeine metabolism | 29 | 29 |
| 144 | Phosphonate and phosphinate metabolism | 28 | 27 |
| 145 | Mismatch repair | 28 | 25 |
| 146 | Aldosterone-regulated sodium reabsorption | 26 | 27 |
| 147 | RIG-I-like receptor signaling pathway | 25 | 25 |
| 148 | Zeatin biosynthesis | 24 | 23 |
| 149 | Glycosaminoglycan biosynthesis - heparan sulfate | 23 | 23 |
| 150 | Two-component system | 18 | 20 |
| 151 | Phenylalanine, tyrosine and tryptophan biosynthesis | 18 | 16 |
| 152 | Non-homologous end-joining | 17 | 20 |
| 153 | Glycosphingolipid biosynthesis - globo series | 17 | 16 |
| 154 | Biotin metabolism | 17 | 16 |
| 155 | Glycosaminoglycan biosynthesis - chondroitin sulfate | 16 | 17 |
| 156 | Taurine and hypotaurine metabolism | 15 | 16 |
| 157 | Geraniol degradation | 15 | 15 |
| 158 | Fatty acid elongation in mitochondria | 15 | 15 |
| 159 | D-Glutamine and D-glutamate metabolism | 14 | 13 |
| 160 | Sulfur relay system | 14 | 15 |
| 161 | Glycosphingolipid biosynthesis - ganglio series | 13 | 13 |
| 162 | Mucin type O-Glycan biosynthesis | 13 | 12 |
| 163 | Glycosphingolipid biosynthesis - lacto and eolacto series | 8 | 10 |
| 164 | Taste transduction | 8 | 10 |
| 165 | Lipoic acid metabolism | 8 | 8 |
| 166 | Vitamin B6 metabolism | 8 | 7 |
| 167 | Synthesis and degradation of ketone bodies | 7 | 7 |
| 168 | Glycosaminoglycan biosynthesis - keratan sulfate | 7 | 8 |
| 169 | Lysine biosynthesis | 7 | 5 |
| 170 | Thiamine metabolism | 6 | 6 |
| 171 | Plant hormone signal transduction | 5 | 5 |
| 172 | DDT degradation | 4 | 4 |
| 173 | Cell cycle - Caulobacter | 4 | 4 |
| 174 | Butirosin and neomycin biosynthesis | 3 | 4 |
| 175 | Biosynthesis of siderophore group nonribosomal peptides | 3 | 3 |
| 176 | Carotenoid biosynthesis | 3 | 3 |
| 177 | Asthma | 3 | 3 |
| 178 | Intestinal immune network for IgA production | 3 | 3 |
| 179 | Indole alkaloid biosynthesis | 3 | 3 |
| 180 | Chlorocyclohexane and chlorobenzene degradation | 2 | 3 |
| 181 | D-Arginine and D-ornithine metabolism | 2 | 2 |
| 182 | Flavone and flavonol biosynthesis | 2 | 1 |
| 183 | C5-Branched dibasic acid metabolism | 1 | 1 |
| 184 | Toluene degradation | 1 | 1 |
| 185 | Polyketide sugar unit biosynthesis | 1 | 1 |
| 186 | D-Alanine metabolism | 1 | 1 |
