# Supplementary material for: De novo transcriptome and expression profile analyses of the Asian corn borer (Ostrinia furnacalis) reveals relevant flubendiamide response genes
Source: BMC Genomics. 2017 Jan 5;18:20. doi: 10.1186/s12864-016-3431-6 (PMC5217215; doi:10.1186/s12864-016-3431-6)
Supplement: Additional file 8: — Information for the primers used in the qRT-PCR analysis. (DOCX 16 kb) [file 12864_2016_3431_MOESM8_ESM.docx]

**Table S8 Information for primers used in qRT-PCR analysis.**

| Unigene | Sequences (5’-3’) |
| --- | --- |
| Unigene_1173 | F: GTAGTTCCCGCCGATGATGT |
|  | R: GAAGCAGGAGGGTGGTCTTC |
| Unigene_13173 | F: CTTTCACGCCTCTCCATGGT |
|  | R: ACTATTGCTGGGTGTGCGAA |
| Unigene_12340 | F: GTAGTTCCCGCCGATGATGT |
|  | R: GAAGCAGGAGGGTGGTCTTC |
| Unigene_586 | F: GAGCAAAGTGGTAGCCGTCT |
|  | R: GGGTCGAGGAGGAAAGCTTC |
| Unigene_29498 | F: GTTGGTTTTCAGACGGTGGC |
|  | R: GTACCTGTTCACGCTGAGCT |
| Unigene_15941 | F: CCATACAGCTTCCCCAGTCC |
|  | R: CAAAGGAAAATGCCGCGGAA |
| Unigene_3898 | F: CGCTGCTGAATTCAAGAGCG |
|  | R: TTCGTCTTGAGGGCCTTCAG |
| Unigene_25476 | F: CTTGATGGTGGTGACGGAGG |
|  | R: CCACTACAGCTGCTCCATCC |
| Unigene_12898 | F: TGAACCAGCTGCACCATAGG |
|  | R: ACCAGGTTCTGTAATGCGCT |
| Unigene_12321 | F: AGCTGGACCAATGAAGGACA |
|  | R: ATGCATTGTGTTGGCTCCAG |
| Unigene_1911 | F: CTGGTTCTTGGCAGCATCTC |
|  | R: GGAAAGGTGGAGATCATCGC |
| Unigene_3568 | F: TGTCCGCTGAGACCAAGAAC |
|  | R: CACTCCAGGTTTGTCGACGA |
| Unigene_17631 | F: TCGCGCCATACTTCACCTTT |
|  | R: AATCCGGGACCCAGAGATCA |
| Unigene_3432 | F: ACGAAGCTGAGAAGTACCGC |
|  | R: AGTATGGTCTGCTTGTCGGC |
| Unigene_4859 | F: CTAGGGAGCACGACGAGAAC |
|  | R: CCAGTATTCAAGCGAGGCCA |
| Unigene_3900 | F: ATTCCACCACCGATTCCACC |
|  | R: TCAAGAAGCCCGACGAACAG |
| Unigene_6059 | F: CGGCCGAGAAGATTCCTCTC |
|  | R: AGCCGCCATATCGATCGTTT |
| Unigene_2960 | F: GTCCAGTGGTCAGGGTGATC |
|  | R: GATGAAGCAGCTGGAGGAGG |
| Unigene_249 | F: ACGACACTAGTGCCTTTGGA |
|  | R: TGGTCAACAGTGTGCAACAG |
| Unigene_4018 | F: CTGCGTCCGGATGAGACTTA |
|  | R: GGGTCGACATCACCTACATTGT |
| Unigene_2468 | F: TTCTCACGAAGCCTCTGTAC |
|  | R: GTGGTCTATGATGGCAGGAA |
| Unigene_7750 | F: ACCACTAATGCGCAGTTCCA |
|  | R: TGTCATGTAGCGGTTGACCC |
| Unigene_1448 | F: GTCCTTGTACCAGTCCTCGC |
|  | R: TTCTACGTGTGCCTGAACGG |
| Unigene_6772 | F: CCGAAGAGATCCTGGAAGCT |
|  | R: AGGTTGGAGCAAGATTCGGA |
| Unigene_10933 | F: ACGCTCAAGACCCAGAAGAG |
|  | R: ACCGAAATATCCACCACCCG |
| Unigene_5356 | F: GGCAACAACGTGATCTTCCG |
|  | R: CGTGCGCCTTATTGTACAGC |
| Unigene_5215 | F: GTTACCACCACCGTCTCACC |
|  | R: AAGGGAATGTCGCCGTCTTT |
| Unigene_2729 | F: TGCTTTCTGTGACTCCTGCT |
|  | R: TCCTCCGACTTTGGCATGTC |
| β-actin | F: CCTTGATGTCACGCACGATT |
|  | R: TCTACGAAGGTTACGCTCTGC |
